# Sporadic Burkitt Lymphoma Presenting as Acute Pancreatitis, Concurrent Sinusitis, and Enlarged Adenoids

**DOI:** 10.1155/2016/3862175

**Published:** 2016-04-24

**Authors:** Vasudha Mahajan, You-Wen Qian, Brooke Blake, Yesenia Rojas-Khalil, Ravi S. Radhakrishnan, Akila Muthukumar

**Affiliations:** ^1^Department of Pediatrics, University of Texas Medical Branch Hospital, 300 University Boulevard, Galveston, TX 77555-0354, USA; ^2^Department of Pathology, University of Texas Medical Branch Hospital, 300 University Boulevard, Galveston, TX 77555-0354, USA; ^3^Division of Pediatric Surgery, Departments of Surgery and Pediatrics, University of Texas Medical Branch, 301 University Boulevard, Galveston, TX 77555-0353, USA; ^4^Division of Hematology Oncology, Department of Pediatrics, University of Texas Medical Branch Hospital, 300 University Boulevard, Galveston, TX 77555-0354, USA

## Abstract

Pancreatitis and sinusitis as presentations of Burkitt lymphoma are uncommon and rarely described in children. We describe here the case of a child who presented with symptoms suggestive of sinusitis unresponsive to antibiotics, with subsequent development of abdominal symptoms due to pancreatitis. He was eventually diagnosed with Burkitt lymphoma.

## 1. Introduction

Burkitt lymphoma is a highly aggressive B-cell non-Hodgkin lymphoma. The sporadic form commonly affects the ileocecal region of the bowel and presents as an abdominal mass. Involvement of the pancreas is rare and few cases have been reported in children. Burkitt lymphoma of the head and neck primarily presents as lymphadenopathy but presentation as sinusitis is uncommon.

We describe the case of an 8-year-old male who presented with symptoms suggestive of sinusitis and also abdominal symptoms suggestive of pancreatitis. On further imaging, he was found to have nasopharyngeal and abdominal masses due to Burkitt lymphoma.

## 2. Case Report

An eight-year-old male was referred to us for complaints of abdominal pain, emesis, and pancreatic mass noted on CT (computerized tomography) scan. His symptoms had started 7 days prior to admission with diffuse mid epigastric pain and postprandial emesis. He did not have any associated symptoms such as fever, diarrhea, abdominal distention, or weight loss. Previous history was significant for a 3-month course of progressively worsening nasal congestion, snoring, and anosmia. He was diagnosed with sinusitis and was initially treated with Clindamycin followed by trimethoprim-sulfamethoxazole and subsequently with a 10-day course of PO Prednisone (0.25 mg/kg daily) without any improvement of symptoms. On admission, he was afebrile, was hydrated, and was not in acute pain. Physical examination was significant for hyponasal voice, nasal congestion, mouth breathing, and bilateral nontender submandibular and anterior cervical lymphadenopathy. His abdomen was soft, was nondistended, and had no palpable masses or organomegaly but epigastric pain was present on palpation. Initial laboratory tests included an elevated lipase (7500 U/L) and amylase (306 U/L) consistent with acute pancreatitis. He was managed with bowel rest and intravenous fluid hydration. Other initial labs included a complete blood count, liver, and renal function tests which were within normal limits. Peripheral smear showed normocytic, normochromic RBC with normal number and morphology of the neutrophils, lymphocytes, and platelets.

Given his upper airway symptoms, MRI (magnetic resonance image) of the orbit and face was done which revealed a 3.1 cm × 5.8 cm × 3 cm nasopharyngeal mass extending into the sphenoid ethmoidal recess causing airway obstruction (Figure 2). Diffuse thickening of bilateral sphenoid, maxillary, and ethmoid sinuses was appreciated. The nasopharyngeal mass was thought to be enlarged adenoids. These findings were followed up by a nasal endoscopy which showed adenoid hypertrophy, nasal polyposis, and findings consistent with chronic sinusitis.

**Figure 1:**
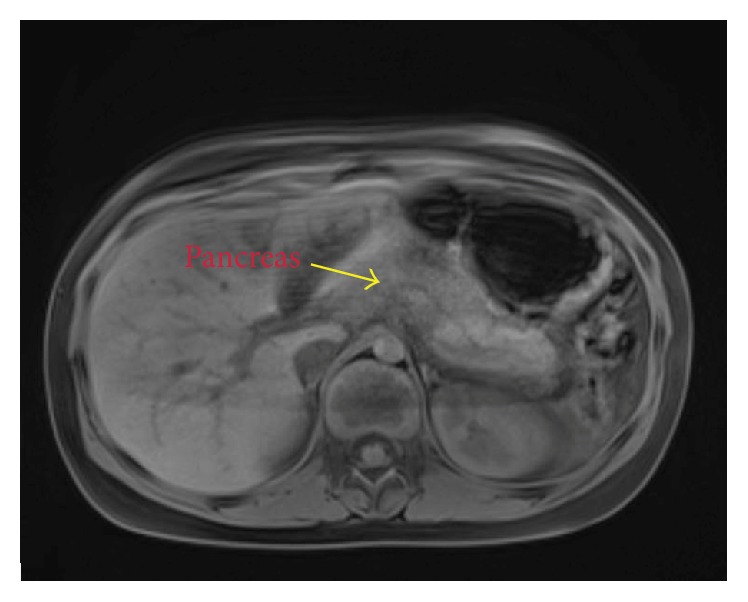
Well-circumcised mass over the head/neck of the pancreas.

**Figure 2:**
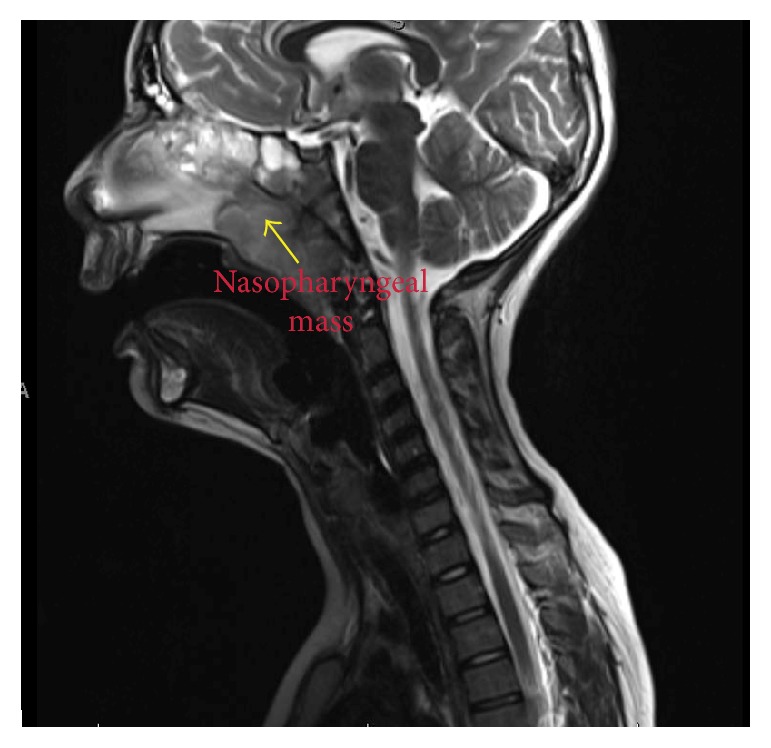
Nasopharyngeal mass causing airway obstruction.

An MRCP (magnetic resonance cholangiopancreatography) was obtained to better characterize the pancreatic mass seen on CT scan. T1 and T2 weighted images revealed a 1.8 cm × 2.1 cm × 1.9 cm well-circumscribed, isointense solid mass over the head and neck of the pancreas (Figure 1). There was mild dilatation of the pancreatic duct proximal to the mass suggesting obstruction. Extra- and intrahepatic biliary ducts were of normal caliber.

After resolution of pancreatitis, patient was taken to the operating room for exploratory laparotomy. A single palpable mass was appreciated at the neck of the pancreas. Intraoperative ultrasound was performed and did not show invasion to the mesenteric vessels. Further examination of the abdomen did not reveal evidence of metastatic disease and hence the mass was deemed resectable. A pancreaticoduodenectomy with classic antrectomy and gastrojejunostomy, pancreaticojejunostomy, and hepaticojejunostomy (Whipple Procedure) was performed. During the creation of the gastrojejunostomy, 2 nodules located on the anterior abdominal wall were palpated. Biopsy of the lesions was submitted to pathology for frozen section and appeared to be “small and blue” in nature; however it could not be further characterized. Wedge resection was performed for cytology evaluation. On gross examination of pancreatic specimen, a smooth, tan, firm lesion measuring 2.1 cm × 2 cm × 2 cm was noted. The pancreatic duct was stenosed, but patent. Margins were negative of disease.

The specimen, as sent for pathology, consisted of a pancreatic head (5 × 3.2 × 2.5 cm), gastric segment (2.5 × 1.8 × 1.5 cm), duodenal segment (30 × 3.2 cm), and gallbladder (7.8 × 5 cm) with common bile duct (5 × 0.2 cm). The pancreatic duct was found stenosed, but patent. Dissection of the specimen revealed a smooth, tan, firm lesion measuring 2.1 × 2 × 2 cm, located 5 cm from the gallbladder neck, bordering the inferior, superior, anterior, and posterior pancreatic margins and extending into the gastric mucosa.

**Figure 3:**
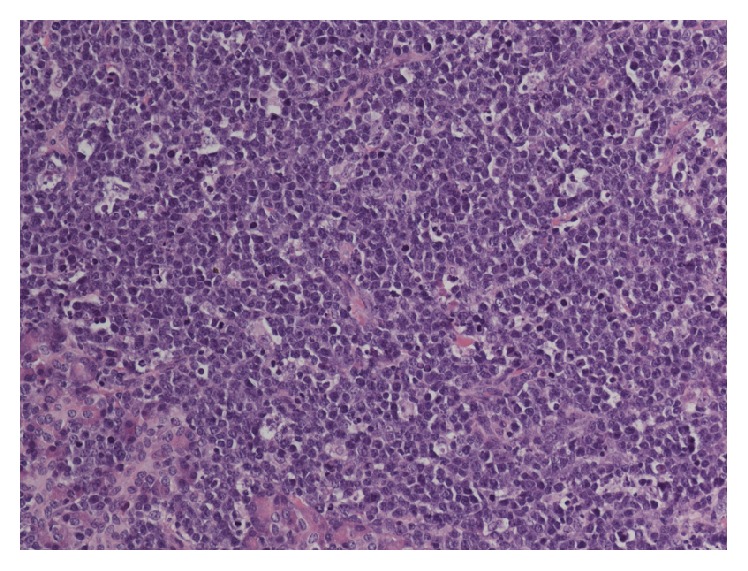
H&E stain of a biopsy specimen from the pancreas showing the characteristic “starry sky appearance” seen in Burkitt lymphoma.

On histologic examination, the majority of the pancreas had been effaced and replaced with sheets of lymphoid infiltrate. The medium-sized cells had a high nucleus : cytoplasm ratio and the round, monomorphic nuclei contained at least one basophilic nucleoli (Figure 3). No tangible body macrophages were visible. The lymphoid cells expressed CD45, CD20, CD10, and BCL-6. They were negative for BCL-2, CD34, and TdT. Cytogenetic testing revealed a c-MYC gene rearrangement; the BCL-2 gene is normal. The cellular activity was measured with Ki-67 and the positivity approaches 100% in the lymphoid cells. CD3 was positive in scattered T cells only. AE1/AE3 was used to detect the presence of epithelial cells, which highlighted the remnants of pancreatic glands. The morphology, cytogenetics, and immunohistochemistry are consistent with Burkitt lymphoma.

Further tests for spread and staging of the lymphoma were done. Bone scan was negative for metastasis. Bilateral bone marrow aspiration showed no increase in blast population and no involvement of bone marrow with lymphoma. Storage iron was absent. c-MYC rearrangement was positive in the tumor tissue but negative for the bone marrow. CSF was positive for malignant lymphoid cells. The patient was stratified under Group C due to his CNS positivity and started on treatment as per standard arm Group C1 under Protocol CCG 5961/FAB LMB 96 protocol [1]. His chemotherapy consisted of a reduction phase of COP (Cyclophosphamide, Vincristine, and Prednisone), followed by multiple cycles including COPADM (Cyclophosphamide, Vincristine, Prednisone, Doxorubicin, IV Methotrexate, IT Methotrexate, and Hydrocortisone) and CYVE (high-dose Cytarabine and Etoposide) with high-dose Methotrexate. PET scan could be done only after three weeks of initiation of chemotherapy which was negative for any metastasis except for inflammatory postsurgical changes.

He had resolution of clinical symptoms after the first cycle of chemotherapy and a repeat MRI showed a significant reduction in the size of the nasopharyngeal mass. This implied that the adenoid enlargement is most likely due to Burkitt lymphoma since there was a remarkable improvement of symptoms immediately after the start of chemotherapy. He is planned to receive maintenance cycles with Methotrexate and Cytarabine.

## 3. Discussion

Acute pancreatitis in children is most commonly secondary to medications like steroids and L-asparaginase, trauma, or anatomical abnormalities in the pancreatic-biliary system [2]. Lymphomas as a cause of pancreatitis is rare. Non-Hodgkins Lymphoma (NHL) accounts for 60% of childhood lymphoma [3]. Of these, only 0.2–2% of them have involvement of the pancreas [4].

Burkitt's lymphoma is an aggressive tumor with a doubling time of 24–48 hours [5]. It is categorized into the endemic, sporadic, and immunodeficiency-associated forms. Histologically, all the forms are similar with high grade, diffuse, and noncleaved B lymphocytes. However, the clinical presentation varies. The endemic form presents as a jaw lesion in 50–70% of the patients. The sporadic form usually presents as an abdominal mass (70–90%) or complications secondary to the mass [3]. Symptoms related to bowel obstruction at initial presentation are common and may be misdiagnosed as appendicitis or intussusception [6]. Approximately 25% of the sporadic form of Burkitt lymphoma involves the head and neck and usually manifests as lymphadenopathy. It is uncommon to have the nasal cavity and paranasal sinuses involved with only a few cases described in adults [5]. It usually involves the maxillary followed by the ethmoid sinuses.

Presentation with bilateral involvement of the maxillary, ethmoid, and sphenoid sinuses, as seen in our patient, is rare. Given its rapid growth, severe facial deformity can result and immediate intervention is mandated. Nasopharyngeal involvement can present with hyponasal speech and otitis media and is frequently misdiagnosed as sinusitis and/or nasal polyposis [7] as was in our patient. Burkitt lymphoma has also been shown to cause direct mass effects on the cranial nerves.

Ultrasound as the initial imaging modality is acceptable for evaluation of the abdominal mass but the extent of the disease, vessel, and lymph node involvement is better delineated with MRI or CT [6]. Positron emission tomography scanning has been shown to identify more abnormalities as compared to CT. The decision on use of PET scan to upstage the disease and modify treatment protocol is still unclear [8].

Due to the high incidence of extranodal disease, the Murphy staging system is used to stage the pediatric lymphomas as compared to the Ann Arbor staging used for adults. Stage IV NHL is defined as tumors involving bone marrow and/or CNS, regardless of other sites of involvement. A patient is considered to have CNS disease if any malignant cell is present in the CSF regardless of cell count. CNS involvement is seen only in 8.8% of the patients with Burkitt lymphoma. The response to first-line therapy is one of the most important prognostic markers for NHL.

## 4. Conclusion

Burkitt lymphoma rarely presents as pancreatitis or sinusitis. Since it is an aggressive tumor with an excellent response to chemotherapy, a high suspicion of Burkitt should be maintained in all children unresponsive to antibiotics and steroids to avoid misdiagnosis and complications.
